# 873. A Retrospective Cohort Study on Treatment Outcomes of Patients on Third-Line Therapy at the HIV Advanced Treatment Centre, University Teaching Hospital, Zambia

**DOI:** 10.1093/ofid/ofab466.1068

**Published:** 2021-12-04

**Authors:** Mona-Gekanju Toeque, Brianna Lindsay, Paul Msanzya Zulu, Sombo Fwoloshi, Duncan Chanda, Francis Mupeta, Mpanji Siwingwa, Melody Mbewe, Lameck Chirwa, Lottie Hachaambwa, Lloyd Mulenga, Cassidy Claassen

**Affiliations:** 1 University of Maryland, Baltimore, Maryland; 2 University of Maryland Medical Center, Baltimore, Maryland; 3 University Teaching Hospital, Lusaka, Lusaka, Zambia

## Abstract

**Background:**

In Zambia, third-line regimens consist of a switch to darunavir/ritonavir (DRV/r) and/or raltegravir (RAL) and/or etravirine (ETV), and as of 2017, dolutegravir (DTG), from a failing second-line therapy.^5^ We assessed virologic suppression (HIV viral load (VL) ≤1000 copies/ml per Zambian national guidelines), immunological response, and patterns of HIV drug resistance mutation among patients on third-line ART at the University Teaching Hospital (UTH) in Lusaka, Zambia.

**Methods:**

A retrospective evaluation of adults ≥18 years old on third-line ART regimens at UTH between January 2012 to June 30, 2020 was conducted. Patients were referred for second-line virologic failure defined as HIV RNA VL > 1000 copies/mL on two consecutive measurements after 6 months on second-line ART.^5^ We assessed virologic suppression VL ≤1000 copies/ml, CD4, mutations, and third-line regimens of this cohort. Patients were excluded if they were on third-line ART < 6 months or received RAL and/or ETV and/or DTG before starting third-line ART.

**Results:**

A total of 539 patients were included; 231 males (42.9%) and 308 (57.1%) females. The mean age of third-line initiation was 29.8 years; mean time from ART initiation to third-line initiation was 9.9 years. Out of 25 combination 349 (64.7%) received DTG, 272 (50.5%) DRV/r, 85(15.8%) ETR, and 49 (9.1%) RAL. There were 215 (39.9%) genotypes; common mutations were to zidovudine (80%), non-nucleoside reverse transcriptase inhibitors (NNRTIs) (78%), and protease inhibitors (PIs) (41%). Patients with at least one viral load and CD4 upon third-line initiation was 296 (54.9%) and 350 (64.9%), respectively. Among patients with sufficient data (21%, n=115), VL suppression increased from 44 (38%) patients at baseline to 53 (46%) at next available follow-up; with mean baseline VL and follow-up VL of log_10_ 3.60 and 3.33, respectively. The immunologic response revealed 49 (56.3%) had CD4 increase with mean increase of 61.1 cells/mm^3^. (See Table 1.)

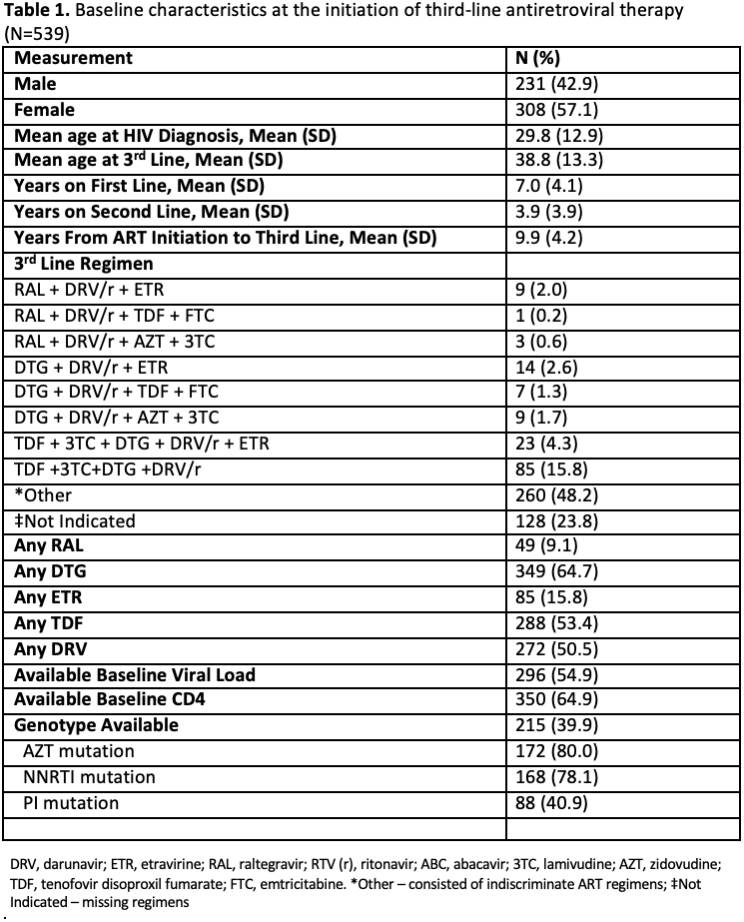

**Conclusion:**

We found moderate improvements in VL suppression and immunologic response. Nearly all third-line patients had genotypic resistance to first-line NNRTI and nearly half to second-line PI regimens. Quality improvement measures are needed to improve viral load timing following ART changes to better assess regimen efficacy.

**Disclosures:**

**All Authors**: No reported disclosures

